# Supplementary material for: Rapid screening of sixty potato cultivars for starch profiles to address a consumer glycemic dilemma
Source: PLoS One. 2023 May 22;18(5):e0255764. doi: 10.1371/journal.pone.0255764 (PMC10202311; doi:10.1371/journal.pone.0255764)
Supplement: S1 File — (DOCX) [file pone.0255764.s001.docx]

The samples of each variety can be purchased through grower catalogs, and Thomas Wagner (author).

The strains names are as followed:

| Bjorna |
| --- |
| Bzura |
| Huckleberry Gold |
| I 1035 |
| Laram K'anchali |
| Muru |
| Olalla |
| Arma |
| Bolivian Blizzard |
| Bareroot River |
| I 1036 |
| Multa |
| Green Mountain |
| Iker |
| Juice Valley |
| Lumper |
| Marble Gold |
| Monona |
| Oct Blue |
| Oct Blue x Col Rose |
| Pirola |
| Biddy Taro |
| Bison |
| C97007 |
| Cherry red |
| Katelidan |
| Norland |
| Papa Amorga |
| Purple Valley |
| Red Pontiac |
| Allegany |
| Anolla |
| Asun |
| Bolivian Spring |
| Russet Burbank |
| Charlotte |
| Chella x Bulk Clover |
| Garnet Chile |
| Golden Anniversary |
| Goldra |
| Leona |
| Mich Oct x John T |
| NH x SPG |
| Nicola |
| Phytophyter |
| Picasso |
| Rush Share |
| Sangre |
| Sassy Lassy |
| Skagit Magic |
| Violet Butter |
| X gem |
| Yukon Gold |
| Arcilla-597779 |
| Chelan |
| Chipitiquilla |
| Dark Red Norland |
| Fontenay |
| Rose Valley |
| Studebaker x Nordic Oct |
