# Supplementary material for: Rapid screening of sixty potato cultivars for starch profiles to address a consumer glycemic dilemma
Source: PLoS One. 2023 May 22;18(5):e0255764. doi: 10.1371/journal.pone.0255764 (PMC10202311; doi:10.1371/journal.pone.0255764)
Supplement: Attachment — Submitted filename: Review_PONE-D-21-22200__HBS_30.7.21.doc [file pone.0255764.s002.doc]

Recommendations:

1. Alpha amylase inhibitory activity (AAIA) of potatoes might have a significant role in lowering Glycemic Index (Low GI) in potatoes so AAIA related enzymatic data should be added to validate the result.
2. All tested potatoes have standard deviation data except Chipitiquilla, Picasso, Chelan, Bzura, Oct Blue. It needs to be explained.
3. In amylose (%) and potential glycemic index equations, R^2^ value should be mentioned with equation obtained.
4. More explanations are needed regarding your calculation of potential glycemic index. You are referring Moreira’s study in 2012 only but need to clarify the adopted equation, logic, condition and justification as you did not conduct any in vivo experiments on GI (AUC method).
5. When denoting correlation between two variables, you have to express in r (Small letter) not r^2^. (GSC score & Amylose and GAC & Amylose). For conducting Pearson’s correlation coefficient tests what is your level of significance?
